# Evaluation of Bioactive Compounds, Minerals and Antioxidant Activity of Lingonberry (*Vaccinium vitis-idaea* L.) Fruits

**DOI:** 10.3390/molecules23010053

**Published:** 2017-12-26

**Authors:** Paulina Dróżdż, Vaida Šėžienė, Józef Wójcik, Krystyna Pyrzyńska

**Affiliations:** 1 Laboratory of the Natural Environment Chemistry, Forest Research Institute, Sękocin Stary, Braci Leśnej 3, 05-090 Raszyn, Poland; J.Wojcik@ibles.waw.pl; 2 Ecology Department, Lithuanian Research Centre for Agriculture and Forestry, Instituto al. 1, Akademija, 58344 Kėdainiai Distr., Lithuania; Vaida.Seziene@mi.lt; 3 Department of Chemistry, University of Warsaw, Pasteura 1, 02-093 Warsaw, Poland; kryspyrz@chem.uw.edu.pl

**Keywords:** lingonberries, polyphenols, flavonoids, anthocyanins, minerals, antioxidant activity

## Abstract

The extraction efficiency of major classes of phenolics from lingonberries grown in the central region of Poland was evaluated. The ethanol–water solution (60:40, *v*/*v*) resulted in the highest extraction yields; however, the results obtained for ethyl acetate were only slightly lower. Total phenolics estimated by Folin-Ciocalteu assay ranged from 468 to 661 mg of GA/100 g fresh weight (fw), while total flavonoids were in the range of 53.2–67.8 μmol/100 g fw. Both solvents exhibited comparable potential for monomeric anthocyanin extraction (26.1–43.0 mg CGE/100 g of fw). The content of several minerals in these fruits and in soil collected from the same places were compared. The essential metal concentrations in all samples increased in the following order: Cr < Cu < Zn < Fe. The levels of toxic elements (Cd, Pb) were acceptable to human consumption for most tested samples. The ethanol-water extracts exhibited the highest scavenging activity against 1,1-diphenyl-2-picryl-hydrazyl (DPPH) radicals, while the highest reducing capacity evaluated by cupric reducing antioxidant capacity (CUPRAC) was obtained for ethyl acetate extracts.

## 1. Introduction

Fresh fruits and derived products provide essential dietary micronutrients and bioactive compounds including vitamins, polyphenols and minerals, with several human health benefits [1]. *Vaccinium vitis-idaea*, also commonly known as lingonberry, partridgeberry or cowberry, is native to boreal forest throughout the Northern Hemisphere from Eurasia to North America. As a member of the *Vaccinium* species, it is related to cranberry, bilberry and blueberry. The berries collected from wild plants growing on publicly accessible lands or bought at the local markets, are a popular fruit in northern, central and eastern Europe. They are consumed raw and cooked in the form of lingonberry jam, compote, juice or syrup. The intake of lingonberry has been associated with anti-inflammatory, antioxidant and antimicrobial activities [2,3,4,5]. Lingonberries exhibited the highest antiproliferative properties among the berries [6]. They have exhibited their therapeutic potential in vitro and in vivo, both in rodent and human models [7].

The wide spectrum of beneficial biological properties of lingoberries is related to their polyphenol constituents, similar to other *Vaccinium* species [8]. Polyphenolic compounds exhibited high antioxidant activity as they can act as reducing agents, hydrogen donors, singlet oxygen quenchers as well as chelators of metal ions, preventing metal catalyzed formation of free radicals [9,10]. Several polyphenolics such as flavonoids, polyphenolic acids, anthocyanins, and procyanidins have been determined in lingonberries [11,12,13,14,15,16]. In addition to these nutrients, they also contain organic acids, vitamins (A, B_1_, B_2_, B_3_ and C), potassium, calcium, magnesium and phosphorous.

The majority of previous studies regarding lingonberries examined fruits collected in the Nordic countries [4,6,12,14,15]. Thus, in this study, the extraction of major classes of polyphenolic compounds from lingonberries grown wild in the central region of Poland was evaluated. The limited area was selected in order to have uniform climatic conditions as well as altitude. Antioxidant activities of the extracts from each plant were evaluated for scavenging ability on 1,1-diphenyl-2-pirylhydrazyl (DPPH) radicals and reducing power by cupric reducing antioxidant capacity (CUPRAC) method. Additionally, the level of several minerals (Cd, Cr, Cu, Fe, Mn, Pb and Zn) in these fruits and in soil collected from the same places was compared.

## 2. Results and Discussion

The review of literature revealed that pure ethanol or methanol extracted polyphenolic compounds with lower efficiency in comparison to hydroalcoholic solutions [17,18,19]. The presence of water is helpful to enhance swelling of plant material, which is favorable to increase the contact surface area between the plant matrix and the solvent, resulting in increase of the extraction yield [18]. Ethyl acetate (EtOAc) has been also successfully applied to extract polyphenolics from berry fruits [17,20]. Thus, in our study water-ethanol mixture (40:60, *v*/*v*) and ethyl acetate were applied.

### 2.1. Total Phenols, Flavonoids and Monomeric Anthocyanins

In order to estimate the main classes of polyphenols present in investigated extracts, three spectrophotometric assays were applied. The content of total phenolics, flavonoids and monomeric anthocyanins in different extracts of lingonberry fruits are given in Table 1. Generally, the use of ethanol-water mixture resulted in improved extraction of target polyphenols; however, the results obtained for EtOAc were only slightly smaller. Total phenolic content in ethanol-water extracts as estimated by FC assay ranged from 468 to 661 mg GAE/100 g fresh weight (fw). The obtained values for sample A (collected in a forest) and D (purchased from the local marketplace) were statistically similar for all studied extracts (*p* < 0.05). For samples B and C (also collected in a forest), higher values were obtained. The polyphenol classification proposed by Vasco et al. [21] using low (<100 mg GAE/100 g), medium (100–500 mg GAE/100 g) and high (>500 mg GAE/100 g) denominations, indicates that our lingonberry samples are a good source of these compounds. According to the data from literature for berries, the obtained results for lingonberries are higher than reported for red raspberries (357.8 mg GAE/100 g) and blueberries (305.4 mg GAE/100 g) [22].

The mean concentration of phenolic compounds in lingonberries grown in the research plot in Oregon (United States) was 566 mg GAE/100 g fw (range 431–660 mg/100 g) [13]. Lower results (in the range of 21.4–24.0 µmole/GAE/100 g fw; it means 36.4–40.8 mg GAE/100 g) for various lingonberry extracts were obtained for fruits harvested from the southern Labrador area in Canada [5]. This discrepancy may be due to the genotype of the plants, as well as geographical location and environmental factors.

Total flavonoids (expressed as catechin equivalent) were in the range of 53.2–67.8 μmole CE/100 g fw for ethanol-water mixture and 40.7–62.9 μmole CE/100 g for ethyl acetate solvent. The flavonoid content of 19.3 ± 0.4 μmole CE/100 g fw in lingonberry fruits collected in Canada was reported [5], while the value of 130.8 mg CE/100 g of dry weight (dw) (450.6 μmole CE/100 g dw) for these berries common in the Nordic diet extracted with 50% aqueous methanol [14]. As reported in the literature, the main flavonoids in lingonberry extracts are flavan-3-ols such as catechin and epicatechin as well as flavonols, mainly quercetin glycosides [14,23].

Table 1 summarizes the total monomeric anthocyanin contents using both solvents. Unlike the total phenolic content, ethanol-water mixture as well as EtOAc exhibited comparable potential for anthocyanin extraction (except for sample A). The obtained results for these compounds were statistically similar (*p* < 0.05) and were in the range of 26.1–43.0 mg CGE/100 g of fresh fruits. The forest samples contained the highest total monomeric anthocyanin contents in comparison to the local marketplace sample. Similar results were obtained for lingonberry fruits collected from a research plot in Oregon [13] and in Canada [5].

### 2.2. Metals

The content of trace metals, which are also the important components of lingonberry fruits, was rarely reported [24,25]. They play an important role in the functioning of the human body as they act as cofactors for various enzymatic systems or possess regulatory activity [26]. Studies for monitoring the concentration of nutrients and potentially toxic elements in food are important to indicate what consumers ingest. Thus, the content of selected metals in lingonberry fruits was determined and the results are shown in Table 2.

In general, the essential metal concentrations in all samples increased in the following order: Cr < Cu < Zn < Fe. The cooper content was statistically similar in the studied samples (*p* < 0.05), except the sample D (purchased from the local marketplace), where significantly higher value (1.14 mg/kg) was determined in comparison to other fruits collected in a forest region. That sample contained also the lowest amount of chromium. The concentrations of toxic Cd was very low (0.001–0.009 mg/kg) and below the maximum allowed limits (0.05 mg/kg) specified by the European Food Legislation [27]. The content of toxic lead was in the range of 0.14–0.17 mg/kg, which also does not exceed the maximum safety value of 0.2 mg/kg defined for this element in fruits [28]. However, the highest amount of Pb was determined in the sample A (0.48 mg/kg), which exceeds the allowed limit.

The content of the metals in fruits is often correlated with their soil content [28]. The transfer of metals from soils to roots and then to other parts of plant is influenced by several factors such as soil properties, environmental conditions, type of metal, plant physiology and rhizosphere biochemistry. Table 3 shows the content of metals in the soil samples collected from the same places as lingonberry fruits. The soils revealed the lowest levels in cadmium (0.041–0.073 mg/kg) and the highest levels in iron (1540–4134 mg/kg). However, no significant correlation was found between the metal content of soils and of the fruits grown on them. The results of one-way ANOVA followed by Tukey’s test indicated that the contents of Cr, Cu, Fe and Zn was varied significantly (*p* > 0.05) among all studied samples. The mean transfer factors of studied metals from the soil to fruits, determined as the ratio of their concentration in fruits to that in the soil, was 0.07, 0.03, 0.13, 0.004, 0.04 and 0.44 for Cd, Cr, Cu, Fe, Pb and Zn, respectively. Fe was the least mobile metal, while Zn was the most. The low level of accumulation of iron, chromium and cooper in lingonberry fruits is probably due to their poor mobility inside the plant and accumulation in the underground parts of the plant [29]. Moreover, the high concentration of zinc in the soil is known to have an antagonistic effect on copper uptake.

### 2.3. The Antioxidant Activity

The antioxidant activities in vitro of lingonberry extracts were determined by the 1,1-diphenyl-2-pirylhydrazyl radicals (DPPH) and cupric reducing antioxidant capacity (CUPRAC) assays. DPPH assay measures the ability of the antioxidants to quench DPPH**^·^** radicals mainly by an electron transfer reaction, while CUPRAC method measures the potential of an antioxidant to reduce copper(II)-neocuproine chelate to the blue Cu(I) complex by electron-donating substances under neutral conditions.

**Figure 1:**
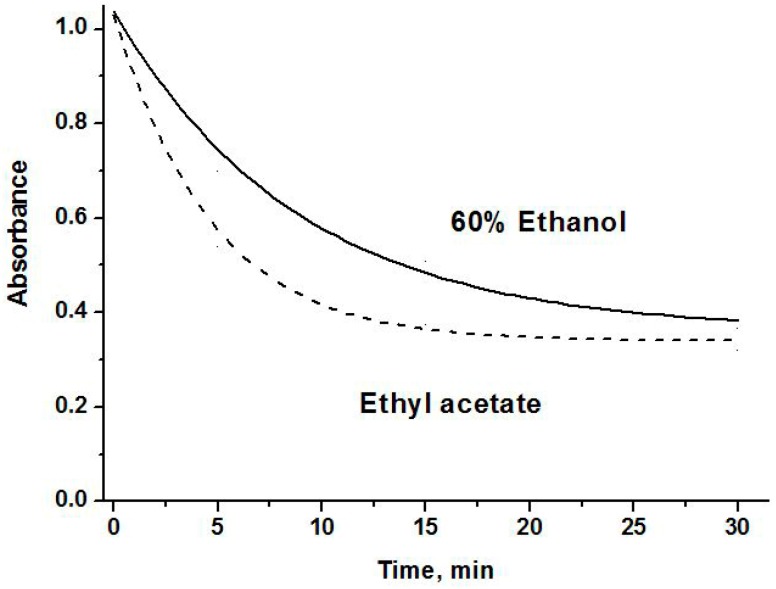
The kinetic curves of scavenged 1,1-diphenyl-2-picryl-hydrazyl (DPPH) radicals by the extracts of lingonberry fruits.

Figure 1 presents the curves based on DPPH bleaching kinetics profiles of ethanol-water and ethyl acetate extracts prepared from fresh lingonberry fruits. The samples collected at location A were taken as the examples. A fast initial decrease of absorbance of DPPH radical followed by slow subsequent disappearance of this reagent can be observed from presented plots. According to Vilaňo et al. [30], this fast step essentially refers to the electron-transfer process from a phenol molecule or its phenoxide anion to DPPH free radicals, while the subsequent decay reflects the remaining activity of the oxidation-degradation products.

**Figure 2:**
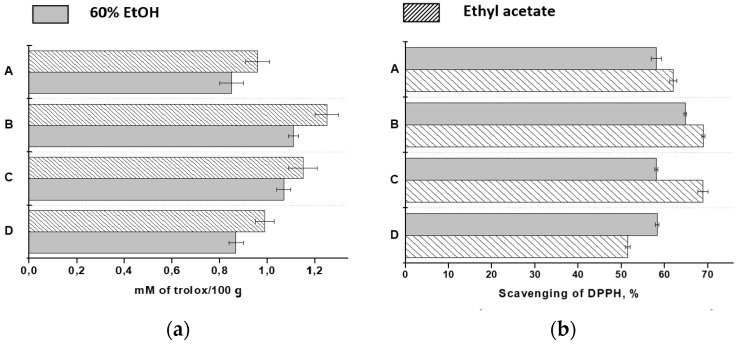
Antioxidant activity of lingonberry extracts evaluated by (**a**) cupric reducing antioxidant capacity (CUPRAC) and (**b**) DPPH assay.

According to the results presented in Figure 2, studied lingonberry fruits are good electron donors as their extracts were able to reduce the cupric complex as well as to quench DPPH radicals. The ethanol-water extracts exhibited higher scavenging activity against DPPH radicals, than ethyl acetate extracts. Similar order was obtained for total phenolics (Table 1). These compounds are responsible for high antioxidant properties of plant extracts. In the CUPRAC method, the highest reducing capacity of examined samples was obtained for ethyl acetate extracts (Figure 2). Probably, this solvent extracts other less polar compounds, which exhibit reducing properties.

Several methods have been applied for evaluation of antioxidant activity in vitro in foodstuff, nutraceuticals and dietary supplements measuring the ability to reduce oxidant species/probes or to scavenge free radicals [31]. However, there is no single, widely-acceptable assay method applicable to a reasonable variety of compounds in different matrices. Very often in order to correlate the results obtained by different methods, a regression model is employed. In some cases, good correlation between the method was reported, despite the difference of assays in the type of radicals used and substrate to be oxidized as well as the basic chemistry in the assays and the biochemistry of the antioxidants. However, in our study very poor correlation between the results obtained by DPPH and CUPRAC assay was obtained (R^2^ = 0.592).

## 3. Materials and Methods

### 3.1. Fruit Samples and Their Extraction

Fruits of wild growing lingonberries were collected at commercial ripeness from pine forests during September of 2016, in three different locations in the Mazovia region. Coordinates for sample A: 52°41′ N, 21°29′ E; sample B: 52°49′ N, 21°45′ E; sample C: 52°01′ N, 21°06′ E. The limited area, central part of Poland was selected, in order to have uniform climatic conditions. Additionally, the sample D was purchased from the local marketplace. Fruits after homogenization by blender were stored at −20 °C until bioactive substances were analyzed.

The homogenized material (700 mg) was shaken with 25 mL ethanol-water (60:40, *v*/*v*) or ethyl acetate for 20 min at room temperature. Then, the extracts were filtered through Whatman no. 1 filter paper. For a given sample, three independent extractions using an appropriate solvent were carried out.

### 3.2. Total Phenolic Content

Total phenolic content of extracts was assessed by using the Folin-Ciocalteu (FC) reagent method [32]. Then, 0.1 mL of extract was mixed with 0.1 mL of FC reagent and 0.9 mL of water. After 5 min, 1 mL of 7% (*w*/*v*) Na_2_CO_3_ and 0.4 mL of water were added. The extracts were mixed and allowed to stand for 30 min before measuring. The absorbance was measured after 30 min at 765 nm using the Lambada Bio 20spectrophotometer (PerkinElmer, Waltham, MA, USA). A mixture of water and reagents was used as a blank. Total phenolic content was expressed as gallic acid equivalents (GAE) in mg/100 g of fresh fruits.

### 3.3. Total Flavonoids

Total flavonoids content was determined using a spectrophotometric method based on formation of flavonoids complex with Al^3+^ ions [33]. First, 1 mL of a sample was mixed with 0.3 mL of NaNO_2_ (5%, *w*/*v*) and 0.5 mL of AlCl_3_ (2%, *w*/*v*). A sample was mixed and six minutes later was neutralized with 0.5 mL of 1 mol/L NaOH solution. The mixture was left for 10 min at room temperature and then absorbance was measured at 510 nm. The results were expressed as catechin equivalent (CE) in µmol per 100 g of fruit.

### 3.4. Total Monomeric Anthocyanins

The total monomeric anthocyanin content was determined using pH-differential method [34]. Absorbance was measured at 520 nm and at 700 nm in buffers at pH 1.0 and 4.5, respectively, using the equation:

*A* = [(*A*_520_ − *A*_700_) _pH1.0_ − (*A*_520_ − *A*_700_) _pH4.5_] (1)

The results were expressed as mg of cyanidin-3-glucoside equivalent (CGE) per 100 g of fresh sample.

### 3.5. Determination of Metals

The samples were digested using the mixture of concentrated HNO_3_ and HClO_4_ acids (4:1 *v*/*v*). First, 0.5 g of fruit sample was placed into 100 mL flask and 10 mL of acid mixture was added into it. This mixture was heated for 30 min at 50 °C on the hot plate. Then, temperature was slowly raised to 160–170 °C and digestion was performed at the beginning for 120 min and finally at 200 °C for 60 min. The digested sample solution was allowed to cool down and transferred into 50 mL volumetric flask diluted by distilled water. The digestion of a reagent blank was performed in parallel.

The content of minerals was determined by inductively coupled plasma atomic emission spectrometry (ICP OES, model iCAP 6000, Thermo Scientific, Waltham, MA, USA) using the following wavelengths (nm): Cd (214.4), Cr (267.7), Cu (224.7), Fe (259.9), Mn (260.5), Pb (220.3) and Zn (206.2) The operating parameters set on the spectrometer were as recommended by manufacturer. The results were expressed in mg/kg of sample fresh weight.

To verify the applicability of the proposed method, standard reference materials Mixed Polish Herbs INCT-MPH-2 and Trace Metals-Silt Clay-1 CRM 045 were utilized. The digestion of the first material was performed with the same decomposition procedure applied for lingonberry samples, where for the digestion of the second material, EPA METHOD 3050B was applied. The obtained results were compared with those provided by manufacturers and are shown in Table 4.

### 3.6. Antioxidant Activity

The DPPH assay was applied to estimate the radical-scavenging ability of the fruit extracts [35]. First, 0.1 mL of a given extract was mixed with 2.4 mL of DPPH solution (9 × 10^−5^ mol/L) in methanol. The change of absorbance at 518 nm was recorded after 30 min. The DPPH scavenging percentage was calculated according to expression (*A*_0_ − *A*_s_)/*A*_0_ × 100, where *A*_0_ is the initial absorbance of DPPH methanolic solution and *A*_s_ is the absorbance of the test sample. 

The cupric ion reducing antioxidant capacity of all extracts was determined according to the method of Apak et al. [36]. The mixture was incubated in a water bath at the temperature of 50 °C for 20 min. The absorbance against the reagent blank was measured after 30 min at 450 nm. The calibration curve was made with trolox and the antioxidant activity was expressed as mM of trolox per gram of fresh fruits.

### 3.7. Statistical Analysis

To verify the statistical significance, mean ± SD of three independent measurements was calculated. Differences between groups were tested by two-way ANOVA followed by Turkey’s test. The differences at *p* < 0.05 were considered significant.

## 4. Conclusions

Lingonberry fruits grown in central Poland are a rich source of dietary polyphenols with strong antioxidant activities. In the current study, ethanol-water (60:40, *v*/*v*) and ethyl acetate solutions provided the highest recovery of different classes of these compounds such as phenolics, flavonoids and anthocyanins. Additionally, the results obtained for trace toxic elements (Cd and Pb) in analyzed fruits were acceptable to human consumption for the most of the tested samples. Lingonberry fruits should be promoted as a dietary source of essential nutrients and health-beneficial compounds and may be among the most important sources of new functional foods in the future.

## Figures and Tables

**Table 1:** Total phenolics, flavonoids and monomeric anthocyanins content in different lingonberry extracts.

Sample	Ethanol–Water (60:40)	Ethyl Acetate
Total Phenolics (mg GAE)		
A	521.0 ± 24.9 ^a^	465.8 ± 29.7 ^a^
B	661.1 ± 22.4 ^b^	594.0 ± 15.1 ^b^
C	654.9 ± 23.0 ^b^	579.8 ± 14.7 ^b^
D	468.0 ± 10.1 ^a^	458.2 ± 22.3 ^a^
Total flavonoids (μg CE)		
A	1.40 ± 0.08 ^a^	1.28 ± 0.08 ^a^
B	1.79 ± 0.09 ^b^	1.49 ± 0.07 ^b^
C	1.12 ± 0.09 ^a^	1.43 ± 0.09 ^c^
D	1.33 ± 0.08 ^c^	1.36 ± 0.09 ^d^
Total monomeric anthocyanins (mg CGE)		
A	43.0 ± 2.87 ^a^	38.1 ± 3.44 ^a^
B	32.1 ± 2.91 ^b^	33.0 ± 2.65 ^a,b^
C	39.2 ± 1.40 ^a^	38.1 ± 2.47 ^a^
D	26.1 ± 1.03 ^b^	26.2 ± 2.19 ^b^

Results expressed as mean ± SD (*n* = 3) per 100 g of fresh weight. Values in each column bearing different superscript letters represent significant differences (*p* < 0.05). GAE—gallic acid equivalent; CE—catechin equivalent; CGE—cyanidin-3-glucose equivalent.

**Table 2:** The content of elements in the lingonberry samples.

Sample	Cd	Cr	Cu	Fe	Pb	Zn
A	0.009 ± 0.0004 ^a^	0.065 ± 0.003 ^a^	0.30 ± 0.015 ^a^	12.4 ± 0.05 ^a^	0.48 ± 0.013 ^a^	4.37 ± 0.17 ^a^
B	0.001 ± 0.00004 ^b^	0.083 ± 0.003 ^b^	0.29 ± 0.012 ^a^	8.25 ± 0.33 ^b^	0.17 ± 0.006 ^b^	5.42 ± 0.22 ^b^
C	0.003 ± 0.0001 ^c^	0.150 ± 0.005 ^c^	0.44 ± 0.021 ^a^	9.93 ± 0.40 ^a^	0.15 ± 0.005 ^b^	4.61 ± 0.16 ^a^
D	0.004 ± 0.0001 ^d^	0.029 ± 0.001 ^d^	1.14 ± 0.046 ^b^	13.4 ± 0.62 ^c^	0.14 ± 0.004 ^b^	4.55 ± 0.14 ^a^

Results expressed in mg/kg of fresh weight as mean ± SD (*n* = 3). Different superscript letters in each column represent significant differences (*p* < 0.05).

**Table 3:** The content of elements in the soil samples.

Sample	Cd	Cr	Cu	Fe	Pb	Zn
A	0.009 ± 0.0004 ^a^	0.065 ± 0.003 ^a^	0.30 ± 0.015 ^a^	12.4 ± 0.05 ^a^	0.48 ± 0.013 ^a^	4.37 ± 0.17 ^a^
B	0.001 ± 0.00004 ^b^	0.083 ± 0.003 ^b^	0.29 ± 0.012 ^a^	8.25 ± 0.33 ^b^	0.17 ± 0.006 ^b^	5.42 ± 0.22 ^b^
C	0.003 ± 0.0001 ^c^	0.150 ± 0.005 ^c^	0.44 ± 0.021 ^a^	9.93 ± 0.40 ^a^	0.15 ± 0.005 ^b^	4.61 ± 0.16 ^a^
D	0.004 ± 0.0001 ^d^	0.029 ± 0.001 ^d^	1.14 ± 0.046 ^b^	13.4 ± 0.62 ^c^	0.14 ± 0.004 ^b^	4.55 ± 0.14 ^a^

Results expressed in mg/kg of fresh weight as mean ± SD (*n* = 3). Different superscript letters in each column represent significant differences (*p* < 0).

**Table 4:** The analysis of certified reference materials (in mg/kg, *n* = 3).

Element	Mixed Polish Herbs INCT-MPH-2		Trace Metals—Silt Clay 1 CRM 045	
	Certified	Determined	Certified	Determined
Cd	0.199 ± 0.015	0.203 ± 0.040	7.01 ± 0.177	7.00 ± 0.071
Cr	1.68 ± 0.12	1.67 ± 0.03	45.7 ± 5.38	47.2 ± 1.87
Cu	7.77 ± 0.53	7.97 ± 0.41	62.2 ± 1.54	62.4 ± 1.09
Fe	460 *	465 ± 7.11	nd	nd
Pb	2.16 ± 0.23	2.16 ± 0.05	45.3 ± 1.92	116 ± 2.17
Zn	33.5 ± 2.1	33.1 ± 0.08	114 ± 6.77	119 ± 1.97

* Information value.

## References

[1] Nile S.H. (2014). Edible berries: Bioactive compounds and their effect on human health. Nutrition.

[2] Wang S.Y., Feng R., Bowman L., Penhallegon R., Ding M., Lu Y. (2005). Antioxidant activity in lingonberries (*Vaccinium vitis-idaea* L.) and its inhibitory effect on activator protein-1, nuclear factor-κB, and mitogen-activated protein kinases activation. J. Agric. Food Chem..

[3] Puišo J., Jonkuviené D., Mačioniené I., Šalomskiené J., Jasutiené I., Kondrotas R. (2014). Biosynthesis of silver nanoparticles using lingonberry and cranberry juices and their antimicrobial activity. Colloids Surf. B.

[4] Kivimäki A.S., Siltari A., Ehlers P.I., Korpel R., Vapaatalo H. (2014). Lingonberry juice negates the effects of a high salt diet on vascular function and low-grade inflammation. J. Funct. Foods.

[5] Bhullar K.S., Rupasinghe H.P.V. (2015). Antioxidant and cytoprotective properties of partridgeberry polyphenols. Food Chem..

[6] Fan Z.L., Wang Z.Y., Liu J.R. (2011). Cold-field fruit extracts exert different antioxidant and antiproliferative activities in vitro. Food Chem..

[7] Su Z. (2012). Anthocyanins and flavonoids of *Vaccinium* L.. Pharm. Crops.

[8] Paredes-López O., Cervantes-Ceja M., Vigna-Pérez M., Hérnàndez-Pérez T. (2010). Berries: Improving human health and healthy aging, and promoting quality life—A review. Plant Food Hum. Nutr..

[9] Galleano M., Verstraeten S.V., Oteiza P.I., Fraga C.G. (2010). Antioxidant actions of flavonoids: Thermodynamic and kinetic analysis. Arch. Biochem. Biophys..

[10] Ghasemzadeh A., Ghasemzadeh N. (2011). Flavonoids and phenolic acids: Role and biochemical activity in plants and humans. J. Med. Plants Res..

[11] Lätti K., Riihinen K.R., Jaakola L. (2011). Phenolic compounds in berries and flowers of a natural hybrid between bilberry and lingonberry (*Vaccinium* × *intermedium* Ruthe). Phytochemistry.

[12] Ek S., Kartimo H., Mattila S., Tolonen A. (2006). Characterization of phenolic compounds from lingonberry (*Vaccinum vitis-idaea*). J. Agric. Food Chem..

[13] Lee J., Finn C.E. (2012). Lingonberry (*Vaccinium vitis*-*idaea* L.) grown in the Pacific Northwest of North America: Anthocyanin and free amino acid composition. J. Funct. Foods.

[14] Hajazimi E., Landberg R., Zamaratskaia G. (2016). Simultaneous determination of flavonols and phenolic acids by HPLC-CoulArray in berries common in the Nordic diet. LWT—Food Sci. Technol..

[15] Tian Y., Liimatainen J., Alanne A.L., Lindstedt A., Liu P., Sinkkonen J., Kallio H., Yang B. (2017). Phenolic compounds extracted by acidic aqueous ethanol from berries and leaves of different berries plants. Food Chem..

[16] Antolak H., Czyżowska A., Sakač M., Mišsan A., Duragič O., Kregiel D. (2017). Phenolic compounds contained in little-known wild fruits as antiadhesive agents against the beverage-spoiling bacteria *Asaia* spp.. Molecules.

[17] Ajila C.M., Brar S.K., Verma M., Tyagi R.D., Godbout S., Valéro J.R. (2011). Extraction and analysis of polyphenols: Recent trends. Crit. Rev. Anal. Chem..

[18] Michiels J.M., Kevers C., Pincemai J., Defraigne J.O., Dommes J. (2012). Extraction conditions can greatly influence antioxidant capacity assays in plant food matrices. Food Chem..

[19] Hwang S.J., Yoon W.B., Lee O.H., Cha S.J., Kim J.D. (2014). Radical-scavenging-linked antioxidant activities of extracts from black chokeberry and blueberry cultivated in Korea. Food Chem..

[20] Bowen-Forbes C.S., Zhang Y., Nair M.G. (2010). Anthocyanin content, antioxidant, anti-inflammatory and anticancer properties of blackberry and raspberry fruits. J. Food Comp. Anal..

[21] Vasco C., Ruales J., Kamal-Eldin A. (2008). Total phenolic compounds and antioxidant capacities of major fruits from Ecuador. Food Chem..

[22] de Souza V.R., Pereira P.A.P., da Silva T.L.T., de Oliveira Lima L.C., Pio R., Queiroz F. (2014). Determination of bioactive compounds, antioxidant activity and chemical composition of Brazilian blackberry, red raspberry, strawberry, blueberry and sweet cherry fruits. Food Chem..

[23] Ieri F., Martini S., Innocenti M., Mulinacci N. (2014). Phenolic distribution in liquid preparations of *Vaccinium myrtillus* L. and *Vaccinium vitis idaea* L.. Phytochem. Anal..

[24] Luginina E.A., Egoshina T.L. (2013). The peculiarities of heavy metals accumulation by wild medicinal and fruit plants. Ann. Warsaw Univ. Life Sci.–SGGW, Ser.: Agric..

[25] Levula T., Saarsalmi A., Rantavaara A. (2000). Effects of ash fertilization and prescribed burning on macronutrients, heavy metal, sulphur and ^137^Cs concentrations in lingonberries (*Vaccinium vitis-idaea*). For. Ecol. Manag..

[26] Fraga C.G. (2005). Relevance, essentiality and toxicity of trace elements in human health. Mol. Aspects Med..

[27] (2008). Regulation (EC) No 629/2008 amending Regulation (EC) No 188/2006 setting maximum levels for certain contaminants in foodstuffs. Off. J. Eur. Union L.

[28] Nowakowska M., Ochmian I., Mijowska K. (2017). The influence of street conditions on sea buckthorn fruit quality and content of micro- and macronutrients in berries and in soil. J. Elem..

[29] He Z., Yang X.E., Stoffellab P.J. (2005). Trace elements in agroecosystems and impacts on the environment. J. Trace Elem. Med. Biol..

[30] Villaño D., Fernández-Pachón M.S., Moyá M.L., Troncoso A.M., García-Parrilla M.C. (2007). Radical scavenging ability of polyphenolic compounds towards DPPH free radical. Talanta.

[31] Apak R., Gorinstein S., Böhm V., Schaich K.M., Őzyürek M., Güçlü K. (2013). Methods of measurement and evaluation of natural antioxidant capacity/activity (IUPAC Technical Report). Pure Appl. Chem..

[32] Singleton V.L., Orhofer R., Lamuela-Raventos R.M. (1999). Analysis of total phenols and other oxidation substrates and antioxidanta by means Folin-Ciocalteu reagent. Methods Enzymol..

[33] Pękal A., Pyrzynska K. (2014). Evaluation of aluminium complexation reaction for flavonoid content assay. Food Anal. Meth..

[34] Lee J., Durst R.W., Wrolstad R.E. (2005). Determination of total monomeric anthocyanin pigment content of fruit juices, beverages, natural colorants, and wines by the pH differential method: Collaborative study. J. AOAC Int..

[35] Pękal A., Pyrzynska K. (2013). Application of free radical diphenylpicrylhydrazyl (DPPH) to estimate the antioxidant capacity of food samples. Anal. Meth..

[36] Apak R., Güçlü K., Özyürek M., Karademir S.E. (2004). Novel total antioxidant capacity index for dietary polyphenols and vitamins C and E, using their cupric ion reducing capability in the presence of neocuproine: CUPRAC method. J. Agric. Food Chem..

